# Functional categorization of gene regulatory variants that cause Mendelian conditions

**DOI:** 10.1007/s00439-023-02639-w

**Published:** 2024-03-04

**Authors:** Y. H. Hank Cheng, Stephanie C. Bohaczuk, Andrew B. Stergachis

**Affiliations:** 1 Division of Medical Genetics, Department of Medicine, University of Washington, Seattle, WA USA; 2 Department of Genome Sciences, University of Washington, Seattle, WA USA; 3 Brotman Baty Institute for Precision Medicine, Seattle, WA USA

## Abstract

Much of our current understanding of rare human diseases is driven by coding genetic variants. However, non-coding genetic variants play a pivotal role in numerous rare human diseases, resulting in diverse functional impacts ranging from altered gene regulation, splicing, and/or transcript stability. With the increasing use of genome sequencing in clinical practice, it is paramount to have a clear framework for understanding how non-coding genetic variants cause disease. To this end, we have synthesized the literature on hundreds of non-coding genetic variants that cause rare Mendelian conditions via the disruption of gene regulatory patterns and propose a functional classification system. Specifically, we have adapted the functional classification framework used for coding variants (i.e., loss-of-function, gain-of-function, and dominant-negative) to account for features unique to non-coding gene regulatory variants. We identify that non-coding gene regulatory variants can be split into three distinct categories by functional impact: (1) non-modular loss-of-expression (LOE) variants; (2) modular loss-of-expression (mLOE) variants; and (3) gain-of-ectopic-expression (GOE) variants. Whereas LOE variants have a direct corollary with coding loss-of-function variants, mLOE and GOE variants represent disease mechanisms that are largely unique to non-coding variants. These functional classifications aim to provide a unified terminology for categorizing the functional impact of non-coding variants that disrupt gene regulatory patterns in Mendelian conditions.

## Introduction

The genetic basis for multiple Mendelian conditions was initially identified by studying individuals harboring chromosomal translocations, which provided a signpost for where in the genome a gene was disrupted. It quickly became apparent that many of these chromosomal translocations did not disrupt coding sequence, but rather disrupted the positioning of coding sequence relative to a distal regulatory element or gene promoter (Vortkamp et al. 1991; Wallis et al. 1999; Fang et al. 2000; Crisponi et al. 2001). These initial studies helped establish that non-coding genetic variation can cause numerous Mendelian conditions, and work over the past several decades has solidified the central role of non-coding genetic variation in the pathogenesis of hundreds of Mendelian conditions.

In this review, we compiled hundreds of non-coding genetic variants from ClinVar and the literature that cause rare human diseases via the disruption of gene regulatory patterns. In doing so, we have recognized that there is no unified vocabulary for describing how this class of genetic variation contributes to Mendelian conditions. Specifically, as is detailed in the section “Functional categorization of gene regulatory variants”, the current functional classification system used for coding variants (i.e., loss-of-function, gain-of-function, and dominant-negative) is not well suited to non-coding variants, because it does not capture the diversity of functional consequences associated with this class of genetic variation. Furthermore, simply describing non-coding variants based on their distance to gene promoters is similarly inadequate. We present a new functional classification system for describing non-coding variants impacting regulatory elements (Table 1) and provide specific examples of variants that fall into each category of this new functional classification system (Tables 2, 3, 4).

**Table 1:** Regulatory variant functional classifications

Functional classification	Definition
Non-modular loss-of-expression (LOE) variants	Genetic variants impacting gene regulatory elements that diminish or completely abolish the intrinsic expression pattern of a gene
Modular loss-of-expression (mLOE) variants	Genetic variants impacting gene regulatory elements that diminish or completely abolish the intrinsic expression pattern of a gene only in a subset of cell types or developmental stages that express that gene
Gain-of-ectopic-expression (GOE) variants	Genetic variants impacting gene regulatory elements that ectopically increase the expression pattern of a gene

**Table 2:** Variants causing LOE

Gene	Disease	OMIM phenotype MIM number	Disease severity	Regulatory element(s) impacted	Distance to promoter	Variant ID	ClinVar ID	ClinVar classification	PMID(s)	Publication date
*ANK1*	Hereditary spherocytosis	182900	Moderate	TATA-binding protein (TBP) and TFIID complex binding element	In promoter	NM_000037.4(ANK1):c.-73_-72del	508	Pathogenic	16037067 (Gallagher et al. 2005)	2005 Sep
*ARPC1B*	Primary immune disorder	617718	Not specified	Not specified	In promoter	9 kb promoter deletion	N/A	N/A	32581362 (Turro et al. 2020)	2020 Jul
*CHRNE*	Congenital myasthenic syndrome	608931	Classical disease	N-box-binding element	In promoter	NM_000080.4(CHRNE):c.-11-85C > T	18353	Likely pathogenic	10211467 (Nichols et al. 1999)	1999 Apr
*CHRNE*	Congenital myasthenic syndrome	608931	Classical disease	N-box-binding element	In promoter	NM_000080.3(CHRNE):c.-95G > A	465866	Pathogenic/likely pathogenic​	11960891 (Abicht et al. 2002)	2002 May
*CHRNE*	Congenital myasthenic syndrome	608931	Classical disease	N-box-binding element	In promoter	NM_000080.3(CHRNE):c.-96G > A	N/A	N/A	10382905 (Ohno et al. 1999)	1999 May
*GALT*	Galactosemia	230400	Duarte variant	E-box-binding element	In promoter	NM_000155.2(GALT):c.-119_-116delGTCA	25111	Pathogenic/likely pathogenic; other​	11286503 (Elsas et al. 2001)	2001 Apr
*GJB1*	Charcot–Marie–Tooth Neuropathy X	302800	Classical disease	Not specified	In promoter	NM_000166.6(GJB1):c.-103C > T	217166	Pathogenic	8757034 (Ionasescu et al. 1996)	1996 Aug
*GJB1*	Charcot–Marie–Tooth Neuropathy X	302800	Classical disease	SOX10-binding element	In promoter	NM_001097642.3(GJB1):c.-16-513 T > C	543921	Likely pathogenic	15470753 (Houlden et al. 2004)	2004 Nov
*GJB1*	Charcot–Marie–Tooth Neuropathy X	302800	Classical disease	SOX10-binding element	In promoter	NM_001097642.3(GJB1):c.-16-513 T > G	10445	Pathogenic	8757034 (Ionasescu et al. 1996)	1996 Aug
*GJB1*	Charcot–Marie–Tooth Neuropathy X	302800	Classical disease	SOX10-binding element	In promoter	NM_001097642.3(GJB1):c.-16-511G > C	10449	Pathogenic	15470753 (Houlden et al. 2004)	2004 Nov
*GJC2*	Pelizaeus-Merzbacher-Like Disease	608804	Milder disease	SOX10-binding element	In promoter	NM_020435.3(GJC2):c.-167A > G	30759	Pathogenic	24374284 (Gotoh et al. 2014)	2014 Mar
*GLIS3*	Diabetes mellitus, neonatal, with congenital hypothyroidism	610199	Milder disease	Not specified	In promoter	426 kb deletion encompassing part of the GLIS3 5' UTR	N/A	N/A	16715098 (Senée et al. 2006)	2006 Jun
*GLIS3*	Diabetes mellitus, neonatal, with congenital hypothyroidism	610199	Milder disease	Not specified	In promoter	134 kb deletion encompassing part of the GLIS3 5' UTR	N/A	N/A	16715098 (Senée et al. 2006)	2006 Jun
*GP1BB*	Bernard–Soulier Syndrome	231200	Classical disease	GATA-binding element	In promoter	NR_037611.1(SEPT5-GP1BB):n.3581C > G	16041	Pathogenic	8703016 (Ludlow et al. 1996)	1996 Sep
*HBB*	Beta-thalassemia	613985	Beta-thalassemia intermedia	CACCC box	In promoter	NM_000518.5(HBB):c.-136C > T	36284	Likely pathogenic	11857738; 28385923 (Hardison et al. 2002; Ropero et al. 2017)	2002 Mar 2017 Oct
*HBB*	Beta-thalassemia	613985	Beta-thalassemia intermedia	CACCC box	In promoter	NM_000518.5(HBB):c.-136C > G	15465	Pathogenic/likely pathogenic​	11857738; 28385923 (Hardison et al. 2002; Ropero et al. 2017)	2002 Mar 2017 Oct
*HBB*	Beta-thalassemia	613985	Beta-thalassemia intermedia	CACCC box	In promoter	NM_000518.5(HBB):c.-137C > G	15464	Pathogenic/likely pathogenic​	11857738; 28385923 (Hardison et al. 2002; Ropero et al. 2017)	2002 Mar 2017 Oct
*HBB*	Beta-thalassemia	613985	Beta-thalassemia intermedia	CACCC box	In promoter	NM_000518.5(HBB):c.-137C > A	36285	Pathogenic/likely pathogenic​	11857738; 28385923 (Hardison et al. 2002; Ropero et al. 2017)	2002 Mar 2017 Oct
*HBB*	Beta-thalassemia	613985	Beta-thalassemia intermedia	CACCC box	In promoter	NM_000518.5(HBB):c.-137C > T	36287	Pathogenic	11857738; 28385923 (Hardison et al. 2002; Ropero et al. 2017)	2002 Mar 2017 Oct
*HBB*	Beta-thalassemia	613985	Beta-thalassemia intermedia	CACCC box	In promoter	NM_000518.5(HBB):c.-138C > A	393701	Pathogenic/likely pathogenic​	11857738; 28385923 (Hardison et al. 2002; Ropero et al. 2017)	2002 Mar 2017 Oct
*HBB*	Beta-thalassemia	613985	Beta-thalassemia intermedia	CACCC box	In promoter	NM_000518.5(HBB):c.-138C > T	15460	Pathogenic	11857738; 28385923 (Hardison et al. 2002; Ropero et al. 2017)	2002 Mar 2017 Oct
*HBB*	Beta-thalassemia	613985	Beta-thalassemia intermedia	CACCC box	In promoter	NM_000518.5(HBB):c.-140C > T	15514	Pathogenic	11857738; 28385923 (Hardison et al. 2002; Ropero et al. 2017)	2002 Mar 2017 Oct
*HBB*	Beta-thalassemia	613985	Beta-thalassemia intermedia	CACCC box	In promoter	NM_000518.5(HBB):c.-142C > T	15462	Pathogenic	11857738; 28385923 (Hardison et al. 2002; Ropero et al. 2017)	2002 Mar 2017 Oct
*HBB*	Beta-thalassemia	613985	Beta-thalassemia intermedia	CACCC box	In promoter	NM_000518.5(HBB):c.-151C > T	15461	Pathogenic	11857738; 28385923 (Hardison et al. 2002; Ropero et al. 2017)	2002 Mar 2017 Oct
*HBB*	Beta-thalassemia	613985	Beta-thalassemia intermedia	TATA box	In promoter	NM_000518.5(HBB):c.-78A > C	15470	Pathogenic	11857738; 28385923 (Hardison et al. 2002; Ropero et al. 2017)	2002 Mar 2017 Oct
*HBB*	Beta-thalassemia	613985	Beta-thalassemia intermedia	TATA box	In promoter	NM_000518.5(HBB):c.-78A > G	15471	Pathogenic/likely pathogenic​	11857738; 28385923 (Hardison et al. 2002; Ropero et al. 2017)	2002 Mar 2017 Oct
*HBB*	Beta-thalassemia	613985	Beta-thalassemia intermedia	TATA box	In promoter	NM_000518.5(HBB):c.-79A > C	869288	Pathogenic	11559932 (Sadiq et al. 2001)	2001 Sep
*HBB*	Beta-thalassemia	613985	Beta-thalassemia intermedia	TATA box	In promoter	NM_000518.5(HBB):c.-79A > G	15469	Pathogenic	11857738; 28385923 (Hardison et al. 2002; Ropero et al. 2017)	2002 Mar 2017 Oct
*HBB*	Beta-thalassemia	613985	Beta-thalassemia intermedia	TATA box	In promoter	NM_000518.5(HBB):c.-80 T > G	869289	Pathogenic	26635043 (Henderson et al. 2016)	2015 Dec
*HBB*	Beta-thalassemia	613985	Beta-thalassemia intermedia	TATA box	In promoter	NM_000518.5(HBB):c.-80 T > C	869290	Pathogenic	11857738; 28385923 (Hardison et al. 2002; Ropero et al. 2017)	2002 Mar 2017 Oct
*HBB*	Beta-thalassemia	613985	Beta-thalassemia intermedia	TATA box	In promoter	NM_000518.5(HBB):c.-80 T > A	15467	Pathogenic	11857738; 28385923 (Hardison et al. 2002; Ropero et al. 2017)	2002 Mar 2017 Oct
*HBB*	Beta-thalassemia	613985	Beta-thalassemia intermedia	TATA box	In promoter	NM_000518.5(HBB):c.-81A > G	15466	Pathogenic	11857738; 28385923 (Hardison et al. 2002; Ropero et al. 2017)	2002 Mar 2017 Oct
*HBB*	Beta-thalassemia	613985	Beta-thalassemia intermedia	TATA box	In promoter	NM_000518.5(HBB):c.-82C > A	15492	Pathogenic	11857738; 28385923 (Hardison et al. 2002; Ropero et al. 2017)	2002 Mar 2017 Oct
*HBD*	Delta-thalassemia	N/A	delta( +)-thalassemia	GATA1 binding element	In promoter	NM_000519.3(HBD):c.-126A > T	15085	Pathogenic	12402333 (De Angioletti et al. 2002)	2002 Nov
*HBD*	Delta-thalassemia	N/A	delta( +)-thalassemia	GATA1 binding element	In promoter	NM_000519.3(HBD):c.-127 T > C	15072	Pathogenic	1515647 (Matsuda et al. 1992)	1992 Sep
*HBD*	Delta-thalassemia	N/A	delta( +)-thalassemia	TATA box	In promoter	NM_000519.3(HBD):c.-81A > G	15087	Pathogenic	15921167 (Frischknecht and Dutly 2005)	2004 Nov
*HCFC1*	X-linked intellectual developmental disorder-3	309541	Classical disease	YY1-binding element	In promoter	NM_005334.3(HCFC1):c.-970 T > C	39694	Pathogenic	23000143 (Huang et al. 2012)	2012 Oct
*HNF1A*	MODY3	600496	Not specified	Not specified	In promoter	NM_000545.8(HNF1A):c.-119del	14936	Pathogenic	10649494 (Godart et al. 2000)	2000 Jan
LDLR	Familial hypercholesterolemia	143890	Not specified	SP1-binding element	In promoter	NM_000527.4(LDLR):c.-138del	3745	Likely pathogenic	14616764 (Dedoussis et al. 2003)	2003 Nov
*MLH1*	Lynch syndrome	609310	Classical disease	Not specified	In promoter	NM_000249.4(MLH1):c.-27C > A	89589	Pathogenic	21840485 (Hitchins et al. 2011)	2011 Aug
*MPL*	Thrombocytopenia	604498	Moderate	Not specified	In promoter	GRCh37:chr1:43803414G > A	N/A	N/A	32581362 (Turro et al. 2020)	2020 Jul
*MSH2*	Lynch Syndrome	120435	Classical disease	E1A-F binding element	In promoter	NM_000251.2(MSH2):c.-78_-77del	90494	Likely pathogenic	17894833 (Yan et al. 2007)	2007 Dec
*NIPBL*	Cornelia de Lange syndrome	122470	Mild form	Not specified	In promoter	NM_133433.4(NIPBL):c.-321_-320delinsA	2151	Pathogenic	16799922 (Borck et al. 2006)	2006 Aug
*PIGY*	Hyperphosphatasia with impaired intellectual development syndrome-6	616809	Milder disease	SP1-binding element	In promoter	NM_001042616.2(PIGY):c.-540G > A	222025	Pathogenic	26293662 (Ilkovski et al. 2015)	2015 Nov
*PKLR*	Pyruvate kinase deficiency	266200	Severe disease	GATA1 binding element	In promoter	NM_000298.6(PKLR):c-72A > G	N/A	N/A	11054094 (Manco et al. 2000)	2000 Sep
*PKLR*	Pyruvate kinase deficiency	266200	Severe disease	Not specified	In promoter	NM_000298.6(PKLR):c-83G > C	N/A	N/A	12393511 (Van Wijk et al. 2003)	2003 Feb
*RB1*	Hereditary retinoblastoma	180200	Unilateral retinoblastoma	ATF binding element	In promoter	NM_000321.2(RB1):c.-189G > T	13085	Pathogenic	1881452 (Sakai et al. 1991)	1991 Sep
*RB1*	Hereditary retinoblastoma	180200	Bilateral and unilateral retinoblastoma	SP1-binding element	In promoter	NM_000321.2(RB1):c.-198G > A	13086	Pathogenic	1881452 (Sakai et al. 1991)	1991 Sep
*RB1*	Hereditary retinoblastoma	180200	Not specified	SP1-binding element	In promoter	NM_000321.3(RB1):c.-206_-189del	995907	Likely Pathogenic	28873162 (Mandelker et al. 2017)	2017 Sep
*RBM8A*	Radial aplasia-thrombocytopenia syndrome (Thrombocytopenia Absent Radius Syndrome , TAR)	274000	Milder disease	Not specified	In promoter	NM_005105.5(RBM8A):c.-21G > A	30464	Pathogenic/Likely pathogenic; other​	22366785 (Albers et al. 2012)	2012 Feb
*SH2D1A*	X-linked lymphoproliferative disease	308240	Not specified	CCAAT box	In promoter	NM_002351.5(SH2D1A):c.-10C > T	10906	Pathogenic	9771704 (Coffey et al. 1998)	1998 Oct
*SLC39A4*	Acrodermatitis enteropathica	201100	Classical disease	CCAAT box	In promoter	NM_130849.4(SLC39A4):c.-169A > G	N/A	N/A	https://doi.org/10.1101/2022.09.09.22279746 (Galey et al. 2022)	2022 Sep
*SPINK1*	Juvenile onset chronic pancreatitis	167800	Not specified	Not specified	In promoter	NM_003122.5(SPINK1):c.-191-24G > A	13762	Pathogenic	11355022 (Kaneko et al. 2001)	2001 Feb
*TXNL4A*	Burn–McKeown syndrome	608572	Classical disease	Not specified	In promoter	NC_000018.9:g.77748581_77748614del34	162203	Conflicting interpretations of pathogenicity​	25434003 (Wieczorek et al. 2014)	2014 Dec
*TXNL4A*	Burn–McKeown syndrome	608572	Classical disease	Not specified	In promoter	NC_000018.9(TXNL4A):g.77748604_77748637del34	190413	Pathogenic	25434003 (Wieczorek et al. 2014)	2014 Dec
*UROS*	Congenital erythropoietic porphyria (CEP)	263700	Severe disease	GATA1 binding element	In promoter	NM_000375.3(UROS):c.-203 T > C	3762	Pathogenic	11254675 (Solis et al. 2001)	2001 Mar
*UROS*	Congenital Erythropoietic Porphyria (CEP)	263700	Mild disease	Not specified	In promoter	NM_000375.3(UROS):c.-26-183G > A	3763	Pathogenic	11254675 (Solis et al. 2001)	2001 Mar
*UROS*	Congenital erythropoietic porphyria (CEP)	263700	Mild disease	Not specified	In promoter	NM_000375.3(UROS):c.-26-193C > A	3764	Pathogenic	11254675 (Solis et al. 2001)	2001 Mar
*UROS*	Congenital erythropoietic porphyria (CEP)	263700	Severe disease	CP2-binding element	In promoter	NM_000375.3(UROS):c.-26-197C > A	3765	Pathogenic	11254675 (Solis et al. 2001)	2001 Mar
*VHL*	von Hippel–Lindau disease	193300	Not specified	Not specified	In promoter	NM_000551.3(VHL):c.-75_-55del	166561	Likely Pathogenic	22357542 (Wu et al. 2012)	2012 Apr
Promoter-distal regulatory variants										
* DLX5*	Split hand-split foot malformation (SHFM)	183600	Reduced penetrance	Not specified	850 kb upstream	1 Mb deletion	N/A	N/A	26075025 (Delgado and Velinov 2015)	2015 Jun
* DLX5*	Split hand-split foot malformation (SHFM)	183600	Reduced penetrance	DYNC1I1 exonic enhancers	835 kb upstream	106 kb deletion	N/A	N/A	24459211 (Allen et al. 2014)	2014 Apr
* DLX5*	Split hand-split foot malformation (SHFM)	183600	Not specified	DYNC1I1 exonic enhancers	370 kb upstream	t(2;7)(p25.1;q22) translocation	N/A	N/A	24459211 (Allen et al. 2014)	2014 Apr
* FOXC2*	Lymphedema-distichiasis syndrome	153400	Not specified	Not specified	120 kb downstream	Translocation	N/A	N/A	11078474 (Fang et al. 2000)	2000 Dec
* FOXG1*	Rett syndrome, congenital variant	613454	Severe phenotype	Enhancer	265 kb downstream	Translocation	N/A	N/A	21441262 (Kortüm et al. 2011)	2011 Jun
* FOXL2*	Blepharophimosis-ptosis-epicanthus inversus syndrome	110100	Not specified	Not specified	180 kb upstream	Translocation	N/A	N/A	11175783 (Crisponi et al. 2001)	2001 Feb
* GATA2*	MonoMAC syndrome	614172	Classical phenotype	Enhancer	9.5 kb downstream	28 bp deletion	N/A	N/A	23502222; 22996659 (Johnson et al. 2012; Hsu et al. 2013)	2013 May 2012 Oct
* GATA2*	MonoMAC syndrome	614172	Classical phenotype	ETS binding element	9.5 kb downstream	NM_032638.5(GATA2):c.1017 + 572C > T	566562	Pathogenic/Likely pathogenic​	23502222; 20040766 (Vinh et al. 2010; Hsu et al. 2013)	2013 May 2010 Feb
* GLI3*	Greig syndrome	175700	Classical phenotype	Not specified	10 kb downstream	Translocation	N/A	N/A	1650914 (Vortkamp et al. 1991)	1991 Aug
* HBA*	Alpha-thalassemia	604131	Not specified	Not specified	10 kb upstream	62 kb deletion	N/A	N/A	2364173 (Hatton et al. 1990)	1990 Jul
* HBA*	Alpha-thalassemia	604131	Not specified	HS-40 element	60 kb upstream	Multiple deletions	N/A	N/A	18391781 (Higgs and Wood 2008)	2008 May
* HBB*	Beta-thalassemia	613985	Milder disease	Not specified	50 kb upstream	Translocation	N/A	N/A	6318113 (Kioussis et al. 1983)	1983 Dec
* IRF6*	Van der Woude syndrome (VWS)	119300	Classical phenotype	Disruption of p63 and E47-binding element, and creation of Lef1 binding element	9.7 kb upstream	MCS9.7-350dupA	N/A	N/A	24442519 (Fakhouri et al. 2014)	2014 May
* LRBA*	Autoantibody-mediated pancytopenia	614700	Not specified	CTCF-binding element	53 kb downstream	7.7 kb deletion	N/A	N/A	32581362 (Turro et al. 2020)	2020 Jul
* MAF*	Cataract, anterior segment dysgenesis and microphthalmia	610202	Severe phenotype (associated with unbalanced translocation)	Not specified	1 Mb upstream	Translocation	N/A	N/A	11772997 (Jamieson et al. 2002)	2002 Jan
* PAH*	Phenylketonuria	261600	Not specified	Enhancer	407 bp upstream	NM_001354304.2(PAH):c.-95-4071_-95-313del, 3.7 kb deletion	638	Likely pathogenic	11935335 (Chen et al. 2002)	2002 Mar
* PAX6*	Aniridia	106210	Classical phenotype	Not specified	150 kb downstream	Translocation	N/A	N/A	7795596 (Fantes et al. 1995)	1995 Mar
* PAX6*	Aniridia	106210	Classical phenotype	PAX6 autoregulatory element	150 kb downstream	NM_019040.5(ELP4):c.1143 + 14176C > A	120328	Pathogenic	24290376 (Bhatia et al. 2013)	2013 Dec
* PITX2*	Axenfeld–Rieger syndrome	180500	Not specified	Not specified	10 kb upstream	7.6 Mb deletion	N/A	N/A	20881290 (Volkmann et al. 2011)	2011 Mar
* PITX2*	Axenfeld–Rieger syndrome	180500	Not specified	Not specified	18 kb upstream	Translocation	N/A	N/A	14991915 (Trembath et al. 2004)	2004 Feb
* PITX2*	Axenfeld–Rieger syndrome	180500	Not specified	Not specified	76 kb upstream	Translocation	N/A	N/A	14991915 (Trembath et al. 2004)	2004 Feb
* PLP1*	Spastic paraplegia type 2	312920	Milder disease	Not specified	136 kb downstream	150 kb duplication	N/A	N/A	16374829 (Lee et al. 2006)	2006 Feb
* POU3F4*	X-linked deafness type 3 (DFN3)	304400	Classical phenotype	Not specified	900 kb downstream	Common 8 kb deletion	N/A	N/A	8872461 (De Kok et al. 1996)	1996 Sep
* SERPINH1*	Osteogenesis imperfecta (OI)	613848	Moderate phenotype	Enhancer	2.37 kb upstream	5,274 bp deletion	N/A	N/A	31179625 (Schwarze et al. 2019)	2019 Aug
* SHH*	Holoprosencephaly	142945	Unspecified	Not specified	265 kb upstream	t(2,7)(q31;q36)	N/A	N/A	9254845 (Roessler et al. 1997)	1997 Aug
* SHH*	Holoprosencephaly	142945	Unspecified	SBE2 enhancer	460 kb upstream	C > T variant	N/A	N/A	18836447 (Jeong et al. 2008)	2008 Nov
* SHOX*	Leri–Weill dyschondrosteosis (LWD)	127300	Classical phenotype	Not specified	205 kb downstream	common 29 kb deletion	N/A	N/A	16175500 (Benito-Sanz et al. 2005)	2005 Oct
* SHOX*	Leri–Weill dyschondrosteosis (LWD) and idiopathic short stature (ISS)	127300, 300582	Milder disease	Enhancer	160 kb downstream	NC_000024.9:g.730550_778092del, 47 kb deletion	66087	Pathogenic	22791839 (Benito-Sanz et al. 2012a, b)	2012 Jul
* SIX3*	Holoprosencephaly	157170	Not specified	Not specified	10–200 kb upstream	Translocation	N/A	N/A	10369266 (Wallis et al. 1999)	1999 Jun
* SOX9*	46,XY male-to-female sex reversal and acampomelic form of campomelic dysplasia	114290	Classical phenotype	Not specified	380 kb upstream	1.5 Mb deletion	N/A	N/A	15060123 (Pop et al. 2004)	2004 Apr
* SOX9*	Campomelic dysplasia	114290	Milder disease	Not specified	917–855 kb upstream	t(7;17)(p13;q24)	N/A	N/A	23648064 (Fonseca et al. 2013)	2013 May
* SOX9*	Campomelic dysplasia	114290	Milder disease	Not specified	601–585 kb upstream	t(17;20)(q24.3;q11.2)	N/A	N/A	23648064 (Fonseca et al. 2013)	2013 May
* SOX9*	Campomelic dysplasia	114290	Milder disease	Not specified	50 kb upstream	t(7;17)(q34;q25.1)	N/A	N/A	23648064; 8348155; 8001137 (Tommerup et al. 1993; Wagner et al. 1994; Fonseca et al. 2013)	2013 May 1993 Jun 1994 Dec
* SOX9*	Campomelic dysplasia	114290	Milder disease	Not specified	74–88 kb upstream	t(12;17)(q21.32;q24.3-q25.1)	N/A	N/A	23648064; 7747782; 8789441 (Ninomiya et al. 1995, 1996; Fonseca et al. 2013)	2013 May 1995 Mar 1996 Jan
* SOX9*	Campomelic dysplasia	114290	Not specified	Not specified	88 kb upstream	t(2;17)(q35;q23-q24)	N/A	N/A	23648064; 7990924; 1583645 (Young et al. 1992; Foster et al. 1994; Fonseca et al. 2013)	2013 May 1994 Dec 1992 Apr
* SOX9*	Campomelic dysplasia	114290	Milder disease	Not specified	110–140 kb upstream	t(9;17)	N/A	N/A	23648064; 9724758 (Wunderle et al. 1998; Fonseca et al. 2013)	2013 May 1998 Sep
* SOX9*	Campomelic dysplasia	114290	Milder disease	Not specified	134–142 kb upstream	t(13;17)(q22;q25.1)	N/A	N/A	23648064; 10364523; 8348155; 8566951 (Tommerup et al. 1993; Wirth et al. 1996; Pfeifer et al. 1999; Fonseca et al. 2013)	2013 May 1999 Jul 1993 Jun 1996 Feb
* SOX9*	Campomelic dysplasia	114290	Milder disease	Not specified	173–179 kb upstream	t(1;17)(q42.13;q24.3-q25.1)	N/A	N/A	23648064; 10364523; 8348155; 8566951 (Tommerup et al. 1993; Wirth et al. 1996; Pfeifer et al. 1999; Fonseca et al. 2013)	2013 May 1999 Jul 1993 Jun 1996 Feb
* SOX9*	Campomelic dysplasia	114290	Not specified	Not specified	161 kb upstream	t(5;17)(q23.2;q24)	N/A	N/A	23648064; 21890680 (Sobreira et al. 2011; Fonseca et al. 2013)	2013 May 2011 Oct
*SOX9*	Campomelic dysplasia	114290	Milder disease	Not specified	212–224 kb upstream	t(6;17)(q14;q24)	N/A	N/A	23648064; 10364523; 8566951 (Wirth et al. 1996; Pfeifer et al. 1999; Fonseca et al. 2013)	2013 May 1999 Jul 1996 Feb
* SOX9*	Campomelic dysplasia	114290	Milder disease	Not specified	228–229 kb upstream	t(10;17)(q24;q23)	N/A	N/A	23648064; 10364523 (Pfeifer et al. 1999; Fonseca et al. 2013)	2013 May 1999 Jul
* SOX9*	Campomelic dysplasia	114290	Milder disease	Not specified	288–319 kb upstream	t(5;17)(q13.3;q24.2)	N/A	N/A	23648064; 10364523 (Pfeifer et al. 1999; Fonseca et al. 2013)	2013 May 1999 Jul
* SOX9*	Campomelic dysplasia	114290	Milder disease	Not specified	70–350 kb upstream	inv(17)(q11.2;q24.3-q25.1)	N/A	N/A	23648064; 7666392; 9724758 (Mansour et al. 1995; Wunderle et al. 1998; Fonseca et al. 2013)	2013 May 1995 Jun 1998 Sep
* SOX9*	Campomelic dysplasia	114290	Milder disease	Not specified	375 kb upstream	t(1;17) (q42.1;q24.3)	N/A	N/A	23648064; 17204049 (Leipoldt et al. 2007; Fonseca et al. 2013)	2013 May 2007 Jan
* SOX9*	Campomelic dysplasia	114290	Milder disease	Not specified	380–1869 kb upstream	del(17)(q24.3)	N/A	N/A	23648064; 15060123 (Pop et al. 2004; Fonseca et al. 2013)	2013 May 2004 Apr
* SOX9*	Campomelic dysplasia	114290	Milder disease	Not specified	517–1477 kb upstream	del(17)(q24.3)	39777	Pathogenic	23648064; 19449405 (Lecointre et al. 2009; Fonseca et al. 2013)	2013 May 2009 Jun
* SOX9*	Campomelic dysplasia	114290	Not specified	Not specified	500–4700 kb upstream	t(7;17)(q33;q24)del(17)(q24.2q24.3)	N/A	N/A	23648064; 20453475 (Jakubiczka et al. 2010; Fonseca et al. 2013)	2013 May 2010 May
* SOX9*	Campomelic dysplasia	114290	Milder disease	Not specified	789 kb upstream	t(Y;17)(q11.2;q24.3)	N/A	N/A	23648064; 17204049 (Leipoldt et al. 2007; Fonseca et al. 2013)	2013 May 2007 Jan
* SOX9*	Pierre Robin sequence (PRS) and campomelic dysplasia	261800, 114290	Milder disease	Not specified	899 kb upstream	t(4;17)(q28.3;q24.3)	N/A	N/A	23648064; 15726498 (Velagaleti et al. 2005; Fonseca et al. 2013)	2013 May 2005 Apr
* SOX9*	Pierre Robin sequence (PRS) and campomelic dysplasia	261800, 114290	Not specified	Not specified	932 kb upstream	t(13;17)(q22.1;q22.3)	N/A	N/A	23648064; 15717285 (Hill-Harfe et al. 2005; Fonseca et al. 2013)	2013 May 2005 Apr
* SOX9*	Campomelic dysplasia	114290	Milder disease	Not specified	900 kb upstream	t(17;22)(q25.1;p11.2)	N/A	N/A	23648064; 10364523; 15717285 (Pfeifer et al. 1999; Hill-Harfe et al. 2005; Fonseca et al. 2013)	2013 May 1999 Jul 2005 Apr
* SRY*	46,XY sex reversal	400044	Classical phenotype	Not specified	1.8 kb upstream	25 kb deletion	N/A	N/A	1438307 (McElreavy et al. 1992)	1992 Nov
* TWIST1*	Saethre–Chotzen syndrome	101400	Classical phenotype	Not specified	260 kb downstream	Inversions and translocation	N/A	N/A	14513358 (Cai et al. 2003)	2003 Dec
* UNC13D*	Familial hemophagocytic lymphohistiocytosis 3	608898	Unspecified	ELF1-binding element	< 1 kb downstream	NM_199242.3(UNC13D):c.118-308C > T	533095	Pathogenic/Likely pathogenic	21931115 (Meeths et al. 2011)	2011 Nov

(Galey et al. 2022; Kioussis et al. 1983; Hatton et al. 1990; Sakai et al. 1991; Vortkamp et al. 1991; Matsuda et al. 1992; McElreavy et al. 1992; Young et al. 1992; Tommerup et al. 1993; Foster et al. 1994; Wagner et al. 1994; Mansour et al. 1995; Ninomiya et al. 1995, 1996; Fantes et al. 1995; Ionasescu et al. 1996; Ludlow et al. 1996; Wirth et al. 1996; De Kok et al. 1996; Roessler et al. 1997; Coffey et al. 1998; Wunderle et al. 1998; Nichols et al. 1999; Pfeifer et al. 1999; Ohno et al. 1999; Wallis et al. 1999; Fang et al. 2000; Godart et al. 2000; Manco et al. 2000; Elsas et al. 2001; Kaneko et al. 2001; Sadiq et al. 2001; Solis et al. 2001; Crisponi et al. 2001; Abicht et al. 2002; De Angioletti et al. 2002; Hardison et al. 2002; Jamieson et al. 2002; Chen et al. 2002; Dedoussis et al. 2003; Van Wijk et al. 2003; Cai et al. 2003; Houlden et al. 2004; Pop et al. 2004; Trembath et al. 2004; Benito-Sanz et al. 2005, 2012b; Frischknecht and Dutly 2005; Hill-Harfe et al. 2005; Velagaleti et al. 2005; Gallagher et al. 2005; Lee et al. 2006; Senée et al. 2006; Borck et al. 2006; Leipoldt et al. 2007; Yan et al. 2007; Higgs and Wood 2008; Jeong et al. 2008; Lecointre et al. 2009; Vinh et al. 2010; Jakubiczka et al. 2010; Hitchins et al. 2011; Volkmann et al. 2011; Kortüm et al. 2011; Sobreira et al. 2011; Meeths et al. 2011; Albers et al. 2012; Wu et al. 2012; Johnson et al. 2012; Huang et al. 2012; Fonseca et al. 2013; Hsu et al. 2013; Bhatia et al. 2013; Allen et al. 2014; Fakhouri et al. 2014; Gotoh et al. 2014; Wieczorek et al. 2014; Delgado and Velinov 2015; Ilkovski et al. 2015; Henderson et al. 2016; Mandelker et al. 2017; Ropero et al. 2017; Schulert et al. 2018; Schwarze et al. 2019; Turro et al. 2020)

**Table 3:** Variants causing mLOE

Gene	Disease	OMIM phenotype MIM number	Phenotype associated with gene LOF variants	OMIM phenotype MIM number for LOF	Regulatory element(s) impacted	Distance to promoter	Variant ID	ClinVar ID	ClinVar classification	PMID(s)	Publication date
*APC*	Gastric adenocarcinoma and proximal polyposis of the stomach	619182	Familial Adenomatous Polyposis	175100	YY1-binding element	APC exon 1B promoter	NM_001127511.3(APC):c.-195A > C	264670	Pathogenic	27087319 (Li et al. 2016)	2016 May
*APC*	Gastric adenocarcinoma and proximal polyposis of the stomach	619182	Familial Adenomatous Polyposis	175100	YY1-binding element	APC exon 1B promoter	NM_001127511.2(APC):c.[-125delA;-195A > C]	243004	Pathogenic	27087319 (Li et al. 2016)	2016 May
*APC*	Gastric adenocarcinoma and proximal polyposis of the stomach	619182	Familial Adenomatous Polyposis	175100	YY1-binding element	APC exon 1B promoter	NM_001127511.3(APC):c.-192A > T	243007	Pathogenic	27087319 (Li et al. 2016)	2016 May
*APC*	Gastric adenocarcinoma and proximal polyposis of the stomach	619182	Familial Adenomatous Polyposis	175100	YY1-binding element	APC exon 1B promoter	NM_001127511.3(APC):c.-192A > G	243006	Likely pathogenic	27087319 (Li et al. 2016)	2016 May
*APC*	Gastric adenocarcinoma and proximal polyposis of the stomach	619182	Familial Adenomatous Polyposis	175100	YY1 binding element	APC exon 1B promoter	NM_001127511.3(APC):c.-191 T > C	243005	Pathogenic	27087319 (Li et al. 2016)	2016 May
*APC*	Gastric adenocarcinoma and proximal polyposis of the stomach	619182	Familial Adenomatous Polyposis	175100	YY1-binding element	APC exon 1B promoter	NM_001127511.3(APC):c.-190G > A	243008	Pathogenic	27087319 (Li et al. 2016)	2016 May
*F9*	Hemophilia B Leyden (mild form)	306900	Hemophilia B	306900	C/EBP-binding element	F9 promoter	NM_000133.4(F9):c.-17del	10561	Pathogenic	2342576 (Crossley and Brownlee 1990)	1990 May
*F9*	Hemophilia B Leyden (mild form)	306900	Hemophilia B	306900	C/EBP-binding element	F9 promoter	NM_000133.4(F9):c.-22 T > C	10644	Pathogenic	2004020 (Royle et al., 1991)	1991 Feb
*F9*	Hemophilia B Leyden (mild form)	306900	Hemophilia B	306900	C/EBP-binding element	F9 promoter	NM_000133.4(F9):c.-17A > G	10646	Pathogenic	2342576 (Crossley & Brownlee 1990)	1990 May
*F9*	Hemophilia B Leyden (mild form)	306900	Hemophilia B	306900	Not specified	F9 promoter	NM_000133.3(F9):c.-35G > A	641767	Pathogenic	2388855 (Crossley et al. 1990)	1990 Aug
*GATA1*	Low platelet count and normal RBC parameters	N/A	Severe platelet and RBC abnormalities	300367	Enhancer	6 kb downstream	4 kb deletion	N/A	N/A	32581362 (Turro et al. 2020)	2020 Jul
*PIGM*	Glycosylphosphatidylinositol deficiency	610293	No gene LOF phenotype reported	N/A	SP1-binding element	PIGM promoter	NM_145167.2(PIGM):c.-270C > G	1288	Pathogenic	16767100 (Almeida et al. 2006)	2006 Jul
*PTF1A*	Isolated pancreatic agenesis	615935	Pancreatic and cerebellar agenesis	609069	Developmental pancreatic enhancer	19 kb downstream	7.6 kb deletion	N/A	N/A	24212882 (Weedon et al. 2014)	2014 Jan
*PTF1A*	Isolated pancreatic agenesis	615935	Pancreatic and cerebellar agenesis	609069	Developmental pancreatic enhancer	25 kb downstream	GRCh37:chr10:23508437A > G	N/A	N/A	24212882 (Weedon et al. 2014)	2014 Jan
*PTF1A*	Isolated pancreatic agenesis	615935	Pancreatic and cerebellar agenesis	609069	Developmental pancreatic enhancer	25 kb downstream	GRCh37:chr10:23508363A > G	N/A	N/A	24212882 (Weedon et al. 2014)	2014 Jan
*PTF1A*	Isolated pancreatic agenesis	615935	Pancreatic and cerebellar agenesis	609069	Developmental pancreatic enhancer	25 kb downstream	GRCh37:chr10:23508305A > G	N/A	N/A	24212882 (Weedon et al. 2014)	2014 Jan
*PTF1A*	Isolated pancreatic agenesis	615935	Pancreatic and cerebellar agenesis	609069	Developmental pancreatic enhancer	25 kb downstream	GRCh37:chr10:23508365A > G	N/A	N/A	24212882 (Weedon et al. 2014)	2014 Jan
*PTF1A*	Isolated pancreatic agenesis	615935	Pancreatic and cerebellar agenesis	609069	Developmental pancreatic enhancer	25 kb downstream	GRCh37:chr10:23508446A > C	N/A	N/A	24212882 (Weedon et al. 2014)	2014 Jan
*SHOX*	Idiopathic short stature (ISS)	300582	Leri–Weill dyschondrosteosis	127300	Enhancer	95 kb upstream	286 kb deletion	N/A	N/A	22071895 (Benito-Sanz et al. 2012a, b)	2012 Jan
*SOX9*	Pierre Robin sequence (PRS)	261800	Campomelic dysplasia	114290	Not specified	1.38 Mb upstream	75 kb deletion	N/A	N/A	19234473 (Benko et al. 2009)	2009 Mar
*SOX9*	Pierre Robin sequence (PRS)	261800	Campomelic dysplasia	114290	Craniofacial region enhancer	1.44 Mb upstream	T > C variant	N/A	N/A	19234473 (Benko et al. 2009)	2009 Mar
*SOX9*	Pierre Robin sequence (PRS)	261800	Campomelic dysplasia	114290	Not specified	1.58 Mb upstream	> 319 kb deletion	N/A	N/A	19234473 (Benko et al. 2009)	2009 Mar
*SOX9*	Pierre Robin sequence (PRS)	261800	Campomelic dysplasia	114290	Not specified	1.16 Mb upstream	t(2;17)(q32;q24)	N/A	N/A	19234473 (Benko et al. 2009)	2009 Mar
*SOX9*	Pierre Robin sequence (PRS)	261800	Campomelic dysplasia	114290	Not specified	1.03 Mb upstream	t(5;17)(q15;q24)	N/A	N/A	19234473 (Benko et al. 2009)	2009 Mar
*SOX9*	Pierre Robin sequence (PRS)	261800	Campomelic dysplasia	114290	Proximal mandibular mesenchyme enhancer	1.23 Mb upstream	t(2;17)(q24.1;q24.3)	N/A	N/A	19234473 (Benko et al. 2009)	2009 Mar
*SOX9*	Pierre Robin sequence (PRS)	261800	Campomelic dysplasia	114290	Not specified	1.56 Mb downstream	36 kb deletion	N/A	N/A	19234473 (Benko et al. 2009)	2009 Mar
*TBX5*	Isolated congenital heart disease	N/A	Holt-Oram Syndrome	142900	TAL1 binding element	90 kb downstream	GRCh37:chr12:114704515G > T	N/A	N/A	22543974 (Smemo et al. 2012)	2012 Jul
*UNC13D*	Recurrent Macrophage Activation Syndrome and Systemic Juvenile Idiopathic Arthritis	N/A	Familial hemophagocytic lymphohistiocytosis 3	608898	NF-κB binding element	UNC13D promoter	NM_199242.3(UNC13D):c.117 + 143A > G	1299418	Not provided	29409136 (Schulert et al. 2018)	2018 Jun

(Crossley and Brownlee 1990; Crossley et al. 1990; Royle et al. 1991; Almeida et al. 2006; Benko et al. 2009; Benito-Sanz et al. 2012a; Smemo et al. 2012; Weedon et al. 2014; Li et al. 2016; Turro et al. 2020)

**Table 4:** Variants causing GOE

Gene	Disease	OMIM phenotype MIM number	Phenotype associated with gene LOF or duplication variants	OMIM phenotype MIM number associated with gene LOF or duplication variants	Regulatory element(s) impacted	Mechanism	Variant ID	ClinVar ID	ClinVar classification	PMID(s)	Publication date
*ANKRD26*	Thrombocytopenia 2	188000	No gene LOF phenotype reported	N/A	Not specified	Increased *ANKRD26* expression	NM_014915.3(ANKRD26):c.-134G > A	30853	Pathogenic/Likely pathogenic​	21211618 (Pippucci et al. 2011)	2011 Jan
*ANKRD26*	Thrombocytopenia 2	188000	No gene LOF phenotype reported	N/A	Not specified	Increased *ANKRD26* expression	NM_014915.3(ANKRD26):c.-128G > C	626920	Likely pathogenic	21211618 (Pippucci et al. 2011)	2011 Jan
*ANKRD26*	Thrombocytopenia 2	188000	No gene LOF phenotype reported	N/A	Not specified	Increased *ANKRD26* expression	NM_014915.3(ANKRD26):c.-128G > A	812727	Pathogenic	21211618 (Pippucci et al. 2011)	2011 Jan
*ANKRD26*	Thrombocytopenia 2	188000	No gene LOF phenotype reported	N/A	Not specified	Increased *ANKRD26* expression	NM_014915.3(ANKRD26):c.-128G > T	812728	Likely pathogenic	21211618 (Pippucci et al. 2011)	2011 Jan
*ANKRD26*	Thrombocytopenia 2	188000	No gene LOF phenotype reported	N/A	Not specified	Increased *ANKRD26* expression	NM_014915.3(ANKRD26):c.-127A > G	626942	Pathogenic	21211618 (Pippucci et al. 2011)	2011 Jan
*ANKRD26*	Thrombocytopenia 2	188000	No gene LOF phenotype reported	N/A	Not specified	Increased *ANKRD26* expression	NM_014915.3(ANKRD26):c.-127A > T	626943	Pathogenic/Likely pathogenic​	21211618 (Pippucci et al. 2011)	2011 Jan
*ANKRD26*	Thrombocytopenia 2	188000	No gene LOF phenotype reported	N/A	Not specified	Increased *ANKRD26* expression	NM_014915.3(ANKRD26):c.-127A > C	1175767	Likely pathogenic	21211618 (Pippucci et al. 2011)	2011 Jan
*ANKRD26*	Thrombocytopenia 2	188000	No gene LOF phenotype reported	N/A	Not specified	Increased *ANKRD26* expression	NM_014915.3(ANKRD26):c.-126 T > C	626941	Pathogenic/Likely pathogenic​	21211618 (Pippucci et al. 2011)	2011 Jan
*ANKRD26*	Thrombocytopenia 2	188000	No gene LOF phenotype reported	N/A	Not specified	Increased *ANKRD26* expression	NM_014915.3(ANKRD26):c.-126 T > G	1684447	Pathogenic	21211618 (Pippucci et al. 2011)	2011 Jan
*ANKRD26*	Thrombocytopenia 2	188000	No gene LOF phenotype reported	N/A	Not specified	Increased *ANKRD26* expression	NM_014915.3(ANKRD26):c.-118C > G	626940	Likely pathogenic	21211618 (Pippucci et al. 2011)	2011 Jan
*ANKRD26*	Thrombocytopenia 2	188000	No gene LOF phenotype reported	N/A	Not specified	Increased *ANKRD26* expression	NM_014915.3(ANKRD26):c.-118C > T	627410	Pathogenic/Likely pathogenic​	21211618 (Pippucci et al. 2011)	2011 Jan
*ANKRD26*	Thrombocytopenia 2	188000	No gene LOF phenotype reported	N/A	Not specified	Increased *ANKRD26* expression	NM_014915.3(ANKRD26):c.-118C > A	812726	Likely pathogenic	21211618 (Pippucci et al. 2011)	2011 Jan
*ANKRD26*	Thrombocytopenia 2	188000	No gene LOF phenotype reported	N/A	Not specified	Increased *ANKRD26* expression	NM_014915.3(ANKRD26):c.-116C > G	627189	Likely pathogenic	21211618 (Pippucci et al. 2011)	2011 Jan
*BMP2*	Autosomal-dominant brachydactyly type A2 (BDA2)	112600	LOF variants cause Short Stature, Facial Dysmorphism, and Skeletal Anomalies	617877	Disruption of limb-specific enhancer of BMP2	Not specified	4.6–5.9 kb duplications located 110 kb downstream of BMP2 gene	29614	Pathogenic	19327734; 21357617 (Dathe et al. 2009; Su et al. 2011)	2009 Apr 2011 May
*CTSB*	Keratolytic winter erythema (KWE)	148370	No gene LOF phenotype reported	N/A	Duplicated CTSB enhancer	Increased CTSB expression	7.6 and 15.9 kb duplications located 8 kb upstream of CTSB gene	N/A	N/A	28457472 (Ngcungcu et al. 2017)	2017 May
*CYP11B1*	Glucocorticoid-remediable aldosteronism (GRA)	103900	LOF variants cause congenital adrenal hyperplasia/congenital hypoaldosteronism	202010	Entire CYP11B2 promoter disrupted	Misexpression of CYP11B2 gene under control of CYP11B1 promoter	CYP11B1, CYP11B1/CYP11B2 ANTI-LEPORE-LIKE CHIMERA	1172	Pathogenic	1731223 (Lifton et al. 1992)	1992 Jan
*HBG1*	Hereditary persistence of fetal hemoglobin	141749	No gene LOF phenotype reported	N/A	CCAAT box	Persistence of gamma-globin expression into adulthood	NM_000559.2(HBG1):c.-170G > A	15030	Pathogenic	1379347 (Berry et al. 1992)	1992 Aug
*HBG1*	Hereditary persistence of fetal hemoglobin	141749	No gene LOF phenotype reported	N/A	CCAAT box	Persistence of gamma-globin expression into adulthood	NM_000559.2(HBG1):c.-167C > T	15035	Pathogenic	1704803; 1698280 (Fucharoen et al. 1990; Oner et al. 1991)	1991 Mar 1990 Sep
*HBG1*	Hereditary persistence of fetal hemoglobin	141749	No gene LOF phenotype reported	N/A	ZBTB7A-binding element	Persistence of gamma-globin expression into adulthood via removal of ZBTB7A repression	NM_000559.2(HBG1):c.-53-195C > G	15034	Pathogenic	2224140 (Costa et al. 1990)	1990 Nov
*HBG1*	Hereditary persistence of fetal hemoglobin	141749	No gene LOF phenotype reported	N/A	ZBTB7A-binding element	Persistence of gamma-globin expression into adulthood via removal of ZBTB7A repression	NM_000559.2(HBG1):c.-53-196C > T	15033	Pathogenic	2417646; 2423160; 3033668; 1487421 (Gelinas et al. 1986; Loudianos et al. 1992; Pirastu et al. 1987; Waber et al. 1986)	1986 Feb 1986 Jun 1987 May 1992 Sep
*HBG1*	Hereditary persistence of fetal hemoglobin	141749	No gene LOF phenotype reported	N/A	ZBTB7A-binding element	Persistence of gamma-globin expression into adulthood via removal of ZBTB7A repression	NM_000559.2(HBG1):c.-53-198 T > C	15031	Pathogenic	2430647 (Tate et al. 1986)	1986 Dec
*HBG2*	Hereditary persistence of fetal hemoglobin	141749	No gene LOF phenotype reported	N/A	Not specified	Persistence of gamma-globin expression into adulthood	NM_000184.2(HBG2):c.-255C > G	14982	Pathogenic	6208955 (Collins et al. 1984)	1984 Dec
*HBG2*	Hereditary persistence of fetal hemoglobin	141749	No gene LOF phenotype reported	N/A	Not specified	Persistence of gamma-globin expression into adulthood	NM_000184.2(HBG2):c.-228 T > C	14983	Pathogenic	2441598; 7687855 (Craig et al. 1993; Huang et al. 1987)	1987 Aug 1993 May
*HBG2*	Hereditary persistence of fetal hemoglobin	141749	No gene LOF phenotype reported	N/A	CCAAT box	Persistence of gamma-globin expression into adulthood	NM_000184.2(HBG2):c.-167C > T	14990	Pathogenic	1698280 (Fucharoen et al. 1990)	1990 Sep
*HBG2*	Hereditary persistence of fetal hemoglobin	141749	No gene LOF phenotype reported	N/A	CCAAT box	Persistence of gamma-globin expression into adulthood	NM_000184.2(HBG2):c.-167C > A	15001	Pathogenic	10335983 (Zertal-Zidani et al. 1999)	1999 May
*HBG2*	Hereditary persistence of fetal hemoglobin	141749	No gene LOF phenotype reported	N/A	BCL11A-binding element	Persistence of gamma-globin expression into adulthood via removal of BCL11A repression	c.–117G > A	N/A	N/A	29610478 (Martyn et al. 2018)	2018 Apr
*HBG2*	Hereditary persistence of fetal hemoglobin	141749	No gene LOF phenotype reported	N/A	BCL11A-binding element	Persistence of gamma-globin expression into adulthood via removal of BCL11A repression	c.–114C > T	N/A	N/A	29610478 (Martyn et al. 2018)	2018 Apr
*HBG2*	Hereditary persistence of fetal hemoglobin	141749	No gene LOF phenotype reported	N/A	BCL11A-binding element	Persistence of gamma-globin expression into adulthood via removal of BCL11A repression	c.–114C > A	N/A	N/A	29610478 (Martyn et al. 2018)	2018 Apr
*HBG2*	Hereditary persistence of fetal hemoglobin	141749	No gene LOF phenotype reported	N/A	BCL11A-binding element	Persistence of gamma-globin expression into adulthood via removal of BCL11A repression	c.–114C > G	N/A	N/A	29610478 (Martyn et al. 2018)	2018 Apr
*HBG2*	Hereditary persistence of fetal hemoglobin	141749	No gene LOF phenotype reported	N/A	BCL11A-binding element	Persistence of gamma-globin expression into adulthood via removal of BCL11A repression	13 bp deletion ~ 115 bp upstream of TSS	N/A	N/A	29610478 (Martyn et al. 2018)	2018 Apr
*HBG2*	Hereditary persistence of fetal hemoglobin	141749	No gene LOF phenotype reported	N/A	ZBTB7A-binding element	Persistence of gamma-globin expression into adulthood via removal of ZBTB7A repression	c.–197C > T	N/A	N/A	29610478 (Martyn et al. 2018)	2018 Apr
*HBG2*	Hereditary persistence of fetal hemoglobin	141749	No gene LOF phenotype reported	N/A	ZBTB7A-binding element	Persistence of gamma-globin expression into adulthood via removal of ZBTB7A repression	c.–201C > T	N/A	N/A	29610478 (Martyn et al. 2018)	2018 Apr
*HBG2*	Hereditary persistence of fetal hemoglobin	141749	No gene LOF phenotype reported	N/A	ZBTB7A-binding element	Persistence of gamma-globin expression into adulthood via removal of ZBTB7A repression	c.–202C > T	N/A	N/A	29610478 (Martyn et al. 2018)	2018 Apr
*HBG2*	Hereditary persistence of fetal hemoglobin	141749	No gene LOF phenotype reported	N/A	ZBTB7A-binding element	Persistence of gamma-globin expression into adulthood via removal of ZBTB7A repression	c.–202C > G	N/A	N/A	29610478 (Martyn et al. 2018)	2018 Apr
*IHH*	Polydactyly	N/A	No gene LOF phenotype reported	N/A	CTCF-associated TAD boundary	Duplication brings the IHH/Ihh gene in proximity to the centromeric portion of the EPHA4-containing TAD. This causes IHH ectopic expression in the developing limb, in a pattern resembling endogenous Epha4 expression	~ 900 kb duplication in chromosomal region 2q35	N/A	N/A	25959774 (Lupiáñez et al. 2015)	2015 May
*MMP19*	Cavitary optic disk anomaly (CODA)	611543	No gene LOF phenotype reported	N/A	Enhancer	Increased MMP19 expression	GRCh38/hg38 12q13.2(chr12:55,845,043–55851177) × 4, 6 kb triplication located 2.1 kb upstream of the MMP19	180748	Pathogenic	25581579 (Hazlewood et al. 2015)	2015 Mar
*MTX2/HOXD genes*	Mesomelic dysplasia and vertebral defects	N/A	MTX2 LOF variants cause Mandibuloacral dysplasia progeroid syndrome	619,127	Disruption of distal MTX2 regulatory elements	Not specified	t(2;8)(q31;p21) balanced translocation with breakpoint between MTX2 gene and HOXD gene cluster	N/A	N/A	11944980 (Spitz et al. 2002)	2002 Apr
*OVOL2*	Corneal dystrophy, posterior polymorphous, 1	122000	No gene LOF phenotype reported	N/A	Not specified	Increased *OVOL2* expression	NM_001303461.1(OVOL2):c.-297 + 886 T > C	224838	Pathogenic	26749309 (Davidson et al. 2016)	2016 Jan
*OVOL2*	Corneal dystrophy, posterior polymorphous, 1	122000	No gene LOF phenotype reported	N/A	Not specified	Increased *OVOL2* expression	NM_001303461.1(OVOL2):c.-297 + 895_-297 + 916dup	224837	Pathogenic	26749309 (Davidson et al. 2016)	2016 Jan
*OVOL2*	Corneal dystrophy, posterior polymorphous, 1	122000	No gene LOF phenotype reported	N/A	Not specified	Increased *OVOL2* expression	NM_001303461.1(OVOL2):c.-297 + 949 T > C	224839	Pathogenic	26749309 (Davidson et al. 2016)	2016 Jan
*OVOL2*	Corneal dystrophy, posterior polymorphous, 1	122000	No gene LOF phenotype reported	N/A	Not specified	Increased *OVOL2* expression	NM_021220.4(OVOL2):c.-274 T > G	224840	Pathogenic	26749309 (Davidson et al. 2016)	2016 Jan
*PAX3*	Brachydactyly	N/A	PAX3 LOF variants cause Waardenburg syndrome	193500	CTCF-associated TAD boundary	Deletions remove the predicted boundary between the EPHA4 and PAX3 TADs. This causes PAX3 ectopic expression at the developing limb, in a pattern resembling endogenous Epha4 expression	Deletions of 1.75–1.9 Mb on chromosome 2q35–36	N/A	N/A	25959774 (Lupiáñez et al. 2015)	2015 May
*PITX1*	Homeotic arm-to-leg transformation	186550	PITX1 LOF variants cause congenital clubfoot with or without deficiency of long bones and/or mirror-image polydactyly	119800	Disruption of PITX1 forelimb suppressor/insulator elements	Not specified	134 kb deletion located 269 kb upstream of the PITX1	N/A	N/A	23022097 (Spielmann et al. 2012)	2012 Oct
*PITX1*	Homeotic arm-to-leg transformation	186550	PITX1 LOF variants cause congenital clubfoot with or without deficiency of long bones and/or mirror-image polydactyly	119800	Disruption of PITX1 forelimb suppressor/insulator elements	Not specified	107 kb deletion located 269 kb upstream of the PITX1	N/A	N/A	23022097 (Spielmann et al. 2012)	2012 Oct
*PITX1*	Homeotic arm-to-leg transformation	186550	PITX1 LOF variants cause congenital clubfoot with or without deficiency of long bones and/or mirror-image polydactyly	119800	Disruption of PITX1 forelimb suppressor/insulator elements	Not specified	Translocation with breakpoint 224 kb upstream of the PITX1	N/A	N/A	23022097 (Spielmann et al. 2012)	2012 Oct
*POMP*	Keratosis linearis with ichthyosis congenita and sclerosis keratoderma (KLICK) syndrome	601952	No gene LOF phenotype reported	N/A	Not specified	Switch in transcription start sites for POMP	NM_015932.5(POMP):c.-95del	116	Pathogenic	20226437 (Dahlqvist et al. 2010)	2010 Apr
*SHH*	Laurin–Sandrow syndrome (LSS)	135750	SHH LOF variants cause holoprosencephaly	142945	Sonic hedgehog (SHH) limb enhancer ZPA regulatory sequence (ZRS)	Ectopic SHH expression	NC_000007.13:g.156563856_156610632dup, 46.8 kb duplication encompassing the SHH ZRS	157547	Pathogenic	24456159 (Lohan et al. 2014)	2014 Oct
*SHH*	Laurin–Sandrow syndrome (LSS)	135750	SHH LOF variants cause holoprosencephaly	142945	Sonic hedgehog (SHH) limb enhancer ZPA regulatory sequence (ZRS)	Ectopic SHH expression	NC_000007.13:g.156570780_156646750dup, 76 kb duplication encompassing the SHH ZRS	157548	Pathogenic	24456159 (Lohan et al. 2014)	2014 Oct
*SHH*	Laurin–Sandrow syndrome (LSS)	135750	SHH LOF variants cause holoprosencephaly	142945	Sonic hedgehog (SHH) limb enhancer ZPA regulatory sequence (ZRS)	Ectopic SHH expression	NM_022458.3(LMBR1):c.320-5565_423 + 10975dup, 16.6 kb duplication encompassing the SHH ZRS	157546	Pathogenic	24456159 (Lohan et al. 2014)	2014 Oct
*SHH*	Haas-type polysyndactyly (syndactyly type IV)	186200	SHH LOF variants cause holoprosencephaly	142945	Sonic hedgehog (SHH) limb enhancer ZPA regulatory sequence (ZRS)	Ectopic SHH expression	GRCh37:chr7:156,437,229–156,692,706 duplication, 255 kb duplication encompassing the SHH ZRS	N/A	N/A	24456159 (Lohan et al. 2014)	2014 Oct
*SHH*	Haas-type polysyndactyly (syndactyly type IV)	186200	SHH LOF variants cause holoprosencephaly	142945	Sonic hedgehog (SHH) limb enhancer ZPA regulatory sequence (ZRS)	Ectopic SHH expression	GRCh37:chr7: 156,491,887–156,671,016 duplication, 179 kb duplication encompassing the SHH ZRS	N/A	N/A	24456159 (Lohan et al. 2014)	2014 Oct
*SHH*	Haas-type polysyndactyly (syndactyly type IV)	186200	SHH LOF variants cause holoprosencephaly	142945	Sonic hedgehog (SHH) limb enhancer ZPA regulatory sequence (ZRS)	Ectopic SHH expression	LMBR1, 235-KB DUP, IVS5, duplication encompassing the SHH ZRS	4905	Pathogenic	18417549 (Sun et al. 2008)	2008 Sep
*SHH*	Triphalangeal thumb-polysyndactyly syndrome	174500	SHH LOF variants cause holoprosencephaly	142945	Sonic hedgehog (SHH) limb enhancer ZPA regulatory sequence (ZRS)	Ectopic SHH expression	NG_009240.1:g.(71605_101850)_(134420_151298)dup, duplication encompassing the SHH ZRS	4901	Pathogenic	18417549 (Sun et al. 2008)	2008 Sep
*SHH*	Preaxial polydactyly	174500	SHH LOF variants cause holoprosencephaly	142945	Sonic hedgehog (SHH) limb enhancer ZPA regulatory sequence (ZRS)	Ectopic SHH expression	t(5,7)(q11,q36), translocation breakpoint at the SHH ZRS	N/A	N/A	12032320 (Lettice et al. 2002)	2002 May
*SHH*	Preaxial polydactyly	174500	SHH LOF variants cause holoprosencephaly	142945	Sonic hedgehog (SHH) limb enhancer ZPA regulatory sequence (ZRS)	Ectopic SHH expression	NM_022458.4(LMBR1):c.423 + 5252A > G	4902	Pathogenic	17152067 (Gurnett et al. 2007)	2007 Jan
*SHH*	Preaxial polydactyly	174500	SHH LOF variants cause holoprosencephaly	142945	Sonic hedgehog (SHH) limb enhancer ZPA regulatory sequence (ZRS)	Ectopic SHH expression	NM_022458.4(LMBR1):c.423 + 5134C > G	4903	Pathogenic	17152067 (Gurnett et al. 2007)	2007 Jan
*SHH*	Triphalangeal thumb	174500	SHH LOF variants cause holoprosencephaly	142945	Sonic hedgehog (SHH) limb enhancer ZPA regulatory sequence (ZRS)	Ectopic SHH expression	NM_022458.4(LMBR1):c.423 + 4808 T > C	4906	Pathogenic	18463159 (Furniss et al. 2008)	2008 Aug
*SHH*	Werner mesomelic syndrome (WMS)	N/A	SHH LOF variants cause holoprosencephaly	142945	Sonic hedgehog (SHH) limb enhancer ZPA regulatory sequence (ZRS)	Ectopic SHH expression	NM_022458.4(LMBR1):c.423 + 4917G > C	155921	Pathogenic	19847792 (Wieczorek et al. 2010)	2010 Jan
*SHH*	Werner mesomelic syndrome (WMS)	N/A	SHH LOF variants cause holoprosencephaly	142945	Sonic hedgehog (SHH) limb enhancer ZPA regulatory sequence (ZRS)	Ectopic SHH expression	NM_022458.4(LMBR1):c.423 + 4917G > A	4898	Pathogenic	19847792 (Wieczorek et al. 2010)	2010 Jan
*SHH*	Haas-type polysyndactyly (syndactyly type IV)	186200	SHH LOF variants cause holoprosencephaly	142945	Sonic hedgehog (SHH) limb enhancer ZPA regulatory sequence (ZRS)	Ectopic SHH expression	LMBR1, 73-KB DUP, duplication encompassing the SHH ZRS	155922	Pathogenic	19847792 (Wieczorek et al. 2010)	2010 Jan
*SLC16A1*	Exercise-induced hyperinsulinism (EIHI)	610021	SLC16A1 LOF variants cause Monocarboxylate transporter 1 deficiency	616095	Not specified	Misexpression of SLC16A1 in beta-cells	NM_003051.4(SLC16A1):c.-202G > A	8916	Pathogenic	17701893 (Otonkoski et al. 2007)	2007 Sep
*SLC16A1*	Exercise-induced hyperinsulinism (EIHI)	610021	SLC16A1 LOF variants cause Monocarboxylate transporter 1 deficiency	616095	Not specified	Misexpression of SLC16A1 in beta-cells	NM_003051.3(SLC16A1):c.-391_-390insACGCCGGTCACGTGGCGGGGTGGGG	8917	Pathogenic	17701893 (Otonkoski et al. 2007)	2007 Sep
*SOX9*	46,XX female-to-male sex reversal	278850	SOX9 LOF variants cause campomelic dysplasia	114290	Disruption of distal SOX9 regulatory element	Increased SOX9 expression	178 kb duplication located 600 kb upstream of SOX9	30708	Pathogenic	21208124 (Cox et al. 2011)	2011 Jan
*SOX9*	46,XX female-to-male sex reversal	278850	SOX9 LOF variants cause campomelic dysplasia	114290	Disruption of distal SOX9 regulatory element	Increased SOX9 expression	96 kb triplication 500 kb upstream of SOX9	192386	Pathogenic	21653197 (Vetro et al. 2011)	2011 Oct
*SOX9*	Brachydactyly-anonychia (Cooks syndrome)	106995	SOX9 LOF variants cause campomelic dysplasia	114290	Disruption of distal SOX9 regulatory element	Not specified	1.21–1.96 mb duplications > 0.1 mb upstream of SOX9	N/A	N/A	19639023 (Kurth et al. 2009)	2009 Aug
*SOX9*	46,XX female-to-male sex reversal	278850	SOX9 LOF variants cause campomelic dysplasia	114290	Disruption of distal SOX9 regulatory element	Increased SOX9 expression	t(12;17)(q14.3;q24.3) with translocation break point > 776 upstream of SOX9	N/A	N/A	20082466 (Refai et al. 2010)	2010 Feb
*TERT*	Familial melanoma predisposition syndrome	615134	TERT LOF variants cause dyskeratosis congenita	613989	Ets/TCF binding element	Altered TERT expression	NM_198253.3(TERT):c.-57A > C	242210	Conflicting interpretations of pathogenicity​ Pathogenic(1); Uncertain significance(2)	23348503 (Horn et al. 2013)	2013 Feb
*WNT6*	F-syndrome	N/A	No gene LOF phenotype reported	N/A	CTCF-associated TAD boundary	An inversion and a duplication both result in bringing the centromeric portion of the EPHA4-containing TAD into closer proximity of the WNT6 gene. This causes WNT6 ectopic expression in the developing limb, in a pattern resembling endogenous Epha4 expression	~ 1.1 Mb inversion with telomeric breakpoint located 1.4 Mb away from the EPHA4 gene and centromeric breakpoint located telomeric of WNT6	N/A	N/A	25959774 (Lupiáñez et al. 2015)	2015 May
*WNT6*	F-syndrome	N/A	No gene LOF phenotype reported	N/A	CTCF-associated TAD boundary	An inversion and a duplication both result in bringing the centromeric portion of the EPHA4-containing TAD into closer proximity of the WNT6 gene. This causes WNT6 ectopic expression in the developing limb, in a pattern resembling endogenous Epha4 expression	~ 1.4 Mb duplication with telomeric breakpoint located 1.2 Mb away from the EPHA4 gene and centromeric breakpoint located centromeric of WNT6	N/A	N/A	25959774 (Lupiáñez et al. 2015)	2015 May
Variants mimicking gene duplication events											
* FOXG1*	Rett syndrome, congenital variant	613454	FOXG1 gene duplications cause Rett syndrome, congenital variant	613454	Silencer	Increased FOXG1 expression	0.4 to 2.1 Mb deletions located < 100 kb to 637 kb downstream of FOXG1	N/A	N/A	22739344 (Allou et al. 2012)	2012 Jun
* LMNB1*	Autosomal-dominant adult-onset demyelinating leukodystrophy (ADLD)	169500	LMNB1 duplications cause Autosomal-dominant adult-onset demyelinating leukodystrophy (ADLD)	169500	Not specified	Not specified	660 kb heterozygous deletion located 66 kb upstream of LMNB1	N/A	N/A	25701871 (Giorgio et al. 2014)	2015 Jun
* NR0B1*	46,XY male-to-female sex reversal	300018	NR0B1 gene duplications cause 46,XY male-to-female sex reversal	300018	Not specified	Not specified	257 kb deletion located 11 kb upstream of the NR0B1 gene	N/A	N/A	17503084 (Smyk et al. 2007)	2007 Aug
* PMP22*	Charcot–Marie–Tooth disease, type 1A	118220	PMP22 gene duplications cause Charcot–Marie–Tooth disease, type 1A	118220	Not specified	Not specified	194 kb duplication located 9 kb upstream of PMP22	N/A	N/A	20493460 (Zhang et al. 2010)	2010 Jun
* PMP22*	Charcot–Marie–Tooth disease, type 1A	118220	PMP22 gene duplications cause Charcot–Marie–Tooth disease, type 1A	118220	Not specified	Not specified	186 kb duplication located 34 kb upstream of PMP22	N/A	N/A	20493460 (Zhang et al. 2010)	2010 Jun

(Collins et al. 1984; Gelinas et al. 1986; Tate et al. 1986; Waber et al. 1986; Huang et al. 1987; Pirastu et al. 1987; Costa et al. 1990; Fucharoen et al. 1990; Oner et al. 1991; Berry et al. 1992; Lifton et al. 1992; Loudianos et al. 1992; Craig et al. 1993; Zertal-Zidani et al. 1999; Spitz et al. 2002; Lettice et al. 2002; Gurnett et al. 2007; Smyk et al. 2007; Otonkoski et al. 2007; Furniss et al. 2008; Sun et al. 2008; Dathe et al. 2009; Kurth et al. 2009; Wieczorek et al. 2010; Refai et al. 2010; Dahlqvist et al. 2010; Zhang et al. 2010; Cox et al. 2011; Pippucci et al. 2011; Su et al. 2011; Vetro et al. 2011; Spielmann et al. 2012; Allou et al. 2012; Horn et al. 2013; Lohan et al. 2014; Giorgio et al. 2014; Hazlewood et al. 2015; Lupiáñez et al. 2015; Davidson et al. 2016; Ngcungcu et al. 2017; Martyn et al. 2018)

Of note, non-coding variants can cause Mendelian conditions via a myriad of mechanisms, and this review specifically focuses on rare variants that cause Mendelian conditions by disrupting gene regulatory elements. Other classes of non-coding genetic variants include intronic variants that disrupt transcript splicing, 5’UTR genetic variants that alter initiation codon usage, 3’UTR and/or 5’UTR genetic variants that impact transcript stability, localization, or signal response, genetic variants within non-coding RNAs (ncRNAs), and non-coding repeat expansions that form altered RNA products (Stenson et al. 2017; French and Edwards 2020). Additionally, CpG methylation alterations at imprinted loci cause a handful of Mendelian conditions without altering the underlying DNA sequence (Cerrato et al. 2020). Common non-coding variants associated with common disease risk are covered elsewhere (Zhang and Lupski 2015; Spielmann and Mundlos 2016; French and Edwards 2020).

## Structure of gene regulatory elements

Although the DNA content of a gene is present in every cell of the body, each gene may only be expressed within certain cell types and/or developmental time windows (herein referred to as the ‘intrinsic’ expression pattern of a gene). The intrinsic expression pattern of a gene is governed by regulatory elements, which are short DNA segments (often less than 400 bp in size) containing short binding elements (less than 30 bp) that govern the occupancy of sequence-specific transcription factors (TFs). Regulatory elements are distinguished based on their location relative to the transcriptional start site (TSS) of a gene (i.e., promoter-proximal, vs distal), as well as their functional impact on transcription (i.e., enhancers vs insulators vs silencers).

Promoters overlap the TSS of a gene and contain binding elements (e.g., TATA, CAAT, GC, CACCC boxes, etc.) that modulate RNA polymerase II binding and transcription (Juven-Gershon et al. 2008). Distal regulatory elements have complex roles in regulating the activity of promoters. For example, enhancers upregulate the transcriptional activity of a gene, and can be located adjacent to or over a megabase away from their target gene (Panigrahi and O’Malley 2021). Expression of each gene can be dependent on multiple enhancers, some of which may be common to several cell types. This combinatorial system allows different cell types to express the same gene through overlapping, but distinct, regulatory mechanisms. For example, there are nine different neural enhancers with overlapping spatial expression domains that drive *Sonic Hedgehog* (*SHH*) expression in the brain. Between different brain regions, the set of active enhancers is overlapping, but not identical. This mechanism allows for nuanced control of SHH expression in different parts of the brain (Amano 2020).

Distal regulatory elements can also serve as insulators, which function to compartmentalize adjacent gene regulatory domains along the genome (Gaszner and Felsenfeld 2006). For example, binding of the sequence-specific TF CTCF can create a barrier that limits an enhancer from regulating genes located on the opposite side of a CTCF boundary (Kim et al. 2015). CTCF-bound insulator sequences are often located at the boundaries of topologically associating domains (TADs), which are large chromatin loops (often > 100 kb in size) that enable the creation of transcriptionally independent chromatin domains wherein the activity of regulatory elements is primarily restricted to genes within the same TAD.

In addition, regulatory elements can exhibit context-specific activity. For example, some regulatory elements can act as either enhancers or silencers depending on their cellular context (Erceg et al. 2017; Huang and Ovcharenko 2022). Furthermore, the classification of regulatory elements is actively evolving as we learn how different regulatory elements influence gene expression in different cellular contexts, or in conjunction with neighboring regulatory elements (Ngan et al. 2020). Finally, although the overwhelming majority of variants that impact gene regulation are in the non-coding genome, coding variants can also impact gene regulatory elements (Lango Allen et al. 2014) as ~ 3% of all TF binding elements are located within coding sequences (Stergachis et al. 2013).

## Functional categorization of gene regulatory variants

The functional consequence of coding variants is classified into three distinct categories, loss-of-function (LOF), gain-of-function (GOF), and dominant-negative (DN). LOF variants result in the loss of the normal biological function of a protein via either complete (amorphic) or partial (hypomorphic) LOF. In contrast, GOF variants create a protein with a function distinct from that of the wild-type protein via increasing protein activity (hypermorphic) or creating a completely new function (neomorphic). DN variants create a protein that either directly or indirectly blocks the normal function of the remaining wild-type protein (antimorphic). Notably, both GOF and DN variants are largely defined based on their alterations to the protein product of a gene. In contrast, since gene regulatory variants do not alter the protein sequence of a gene, but rather modulate gene expression patterns, these coding-centric categorizations are often ill suited for gene regulatory variants and create confusion.

**Fig. 1:**
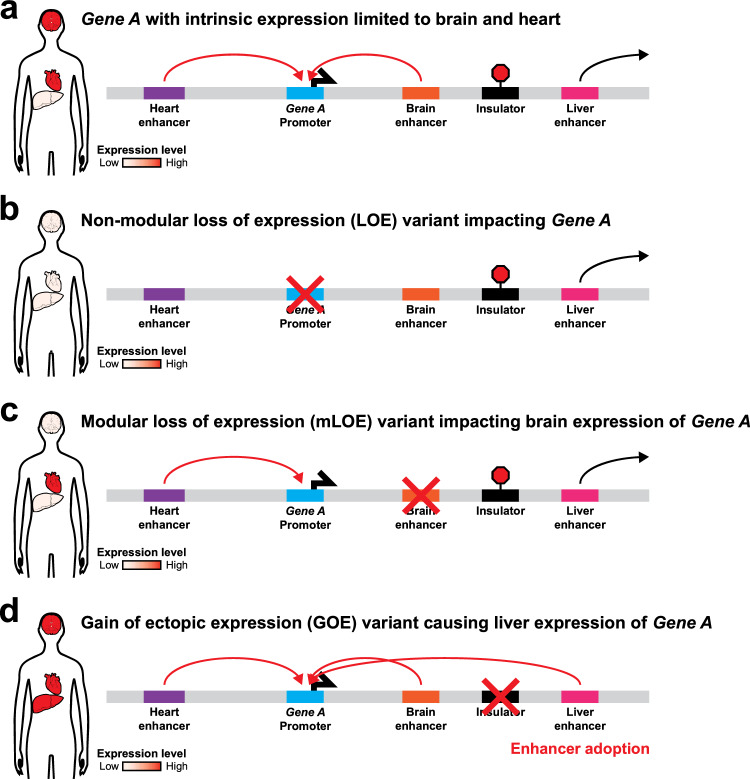
Schematic of the proposed gene regulatory variants functional classifications. **a** Schematic depicting the intrinsic expression pattern of a gene that is limited to the heart and brain, as well as the gene regulatory elements driving this intrinsic expression pattern. **b**–**d** Schematics depicting the impact of **b** LOE, **c** mLOE, and **d** GOE variants on the expression pattern of that gene across different tissues, as well as how various genetic variants can cause these changes

We propose an alternative framework designed to functionally categorize genetic variants that disrupt gene regulatory elements. Specifically, we identify three distinct classes based on their impact on gene regulation at the level of gene transcripts (Table 1): (1) non-modular loss-of-expression (LOE) variants; (2) modular loss-of-expression (mLOE) variants; and (3) gain-of-ectopic-expression (GOE) variants. LOE variants are defined as variants that diminish or completely abolish the expression of a gene universally across all cell types that intrinsically express that gene. In contrast, mLOE variants are defined as variants that diminish or completely abolish the expression of a gene within a limited subset of the cell types or developmental windows that intrinsically express it (i.e., a modular loss of expression). GOE variants are defined as variants that result in the ectopic spatial and/or temporal expression of a gene (Fig. 1). Of note, unlike LOE variants, we chose not to further subdivide GOE variants into modular GOE variants, as it is quite challenging to obtain the appropriate clinical and molecular data that are necessary to firmly state that a GOE variant is truly limited to only a specific developmental window or cell type. For example, with mLOE variants, one can infer that there is a modular loss of expression based on a modular phenotype when compared to coding LOF variants in the same gene, which cause loss of protein function in all tissues or developmental windows. In contrast, with GOE variants, one cannot readily infer from clinical data that the ectopic gain of expression is limited to only a specific cell type. Specifically, a gene product may gain ectopic expression across all cell types, but only have a functional consequence in a select number of cell types—limiting the utility of tissue-selective phenotypes for inferring modular ectopic expression.

As opposed to the traditional LOF, GOF, and DN categories, this functional categorization more intuitively reflects the mechanisms by which disruptions in gene expression patterns cause Mendelian conditions. Of note, these functional classifications can be related to LOF or GOF variant types. For example, LOE variants can correspond to either amorphic or hypomorphic LOF variants. Although mLOE variants could be categorized as hypomorphic LOF variants, the mechanism by which mLOE variants cause disease is quite distinct from that of coding hypomorphic LOF variants, making ‘hypomorphic LOF’ an imprecise label for mLOE variants. In contrast, GOE variants can correspond to hypermorphic GOF, neomorphic GOF, and even LOF variants. Notably, there are no examples of DN gene regulatory variants causing Mendelian conditions in humans. However, this type of regulatory variant has been observed in other organisms and is termed “transvection” (Lewis 1954), which is a phenomenon where a regulatory element on one chromosome interacts with and enhances or silences its corresponding regulatory element on the homologous chromosome. More recently, this mechanism has been described in human cancers, wherein strong enhancers encoded on extrachromosomal circular DNA (ecDNA) can enhance the expression of autosomal genes (Zhu et al. 2021). It is possible that examples of transvection as a cause of Mendelian disease could be described in the future. The LOE, mLOE, and GOE functional categorizations represent the molecular consequences of regulatory variants more closely than the traditional LOF, DN, and GOF classification, and provide an improved framework for conceptualizing the putative role of novel gene regulatory variants in the pathogenesis of Mendelian conditions.

## Non-modular loss-of-expression (LOE) variants

LOE variants diminish or abolish the expression of a gene across all cell types that intrinsically express that gene. Consequently, these variants often mirror the clinical manifestations of coding LOF variants for the same gene, as both LOE and LOF variants result in reduced/absent functional protein levels within the cell, albeit via distinct mechanisms. While some LOE variants cause complete loss of expression (analogous to amorphic LOF variants), others reduce the intrinsic expression level of a gene (analogous to hypomorphic LOF variants). The latter class of variants often results in a more attenuated clinical phenotype compared to variants that result in complete LOE. We have provided examples of over a hundred LOE variants (Table 2), and detail below some key examples of diverse LOE variants.

Many LOE variants are located within the gene promoter, where they disrupt essential TF binding elements required for the intrinsic expression of a gene. For example, genetic variants that disrupt the TATA box and/or CACCC box within the *HBB* gene promoter decrease the intrinsic expression of *HBB* by abrogating the ability of TFs to bind these elements. Notably, these variants do not completely abolish *HBB* transcription, and consequently, individuals harboring these variants in trans with *HBB* LOF variants often still produce adult hemoglobin (HbA), resulting in a milder form of beta-thalassemia (i.e., beta-thalassemia intermedia) compared to individuals with biallelic *HBB* LOF coding variants (Ropero et al. 2017).

Of note, different variants within the same gene promoter can cause varying magnitudes of LOE. For example, variants within the *UROS* promoter that disrupt the GATA1 or CP2-binding elements significantly reduce *UROS* transcription and cause a severe form of congenital erythropoietic porphyria (CEP), whereas other *UROS* promoter variants that do not disrupt these elements only cause a modest reduction in *UROS* transcription and mild cutaneous manifestations (Solis et al. 2001).

LOE variants can also disrupt distal regulatory elements. For example, monocytopenia and mycobacterial infection (MonoMAC) syndrome is typically caused by LOF coding variants within the gene *GATA2*. However, MonoMAC syndrome can also be caused by small deletions or single-nucleotide variants (SNVs) in a *GATA2* intronic enhancer 9.5 kb downstream of the *GATA2* promoter. These variants result in the loss of *GATA2* expression via the disruption of enhancer TF binding elements that are essential for *GATA2* transcription (i.e., an E-box, GATA, and ETS binding element) (Johnson et al. 2012; Hsu et al. 2013).

Variants within regulatory elements that are quite distal to a gene promoter can also cause LOE. For example, most cases of hereditary aniridia are caused by heterozygous LOF coding variants within *PAX6*. However, hereditary aniridia can also be caused by SNVs within a *PAX6* enhancer located 150 kb downstream of the *PAX6* promoter that disrupt a *PAX6* autoregulatory element, causing loss of enhancer activity and subsequent loss of *PAX6* transcription (Bhatia et al. 2013). Furthermore, some patients with hereditary aniridia will have deletions or chromosomal translocations that disrupt this *PAX6* enhancer (Fantes et al. 1995), highlighting the diversity of genetic variant classes that can cause LOE.

Variants that disrupt insulators can also cause LOE. For example, a homozygous deletion of a CTCF-binding site within the first intron of *LRBA* has been reported to cause autoantibody-mediated pancytopenia, a phenotype associated with biallelic coding LOF *LRBA* variants (Turro et al. 2020). It is presumed that loss of this CTCF insulator element alters a TAD boundary, permitting heterochromatin spreading to silence *LRBA* promoter activity.

In summary, LOE variants can be located within promoter or distal gene regulatory elements, can completely mimic coding LOF variants or cause an attenuated phenotype relative to complete LOF variants, and are caused by diverse classes of genetic variants.

## Modular loss-of-expression (mLOE) variants

In contrast to non-modular LOE variants, mLOE variants reduce or abolish the expression of a gene only in a subset of cell types that intrinsically express that gene. mLOE variants represent a disease mechanism largely unique to gene regulatory variants, as coding LOF variants typically disrupt the function of a gene across all cell types that intrinsically express that gene, with the exception of coding LOF variants within exons that are alternatively spliced only within certain tissues or somatic coding LOF variants that only exist within certain tissues (Poduri et al. 2013; Biesecker and Spinner 2013; Jaiswal and Ebert 2019). As a result of their modular impact on gene expression, mLOE variants can produce a subset of features associated with coding LOF variants in that same gene (i.e., phenotype modularity) (Table 3). As gene expression patterns are not typically measured across multiple tissues or developmental stages in individuals with Mendelian conditions, the modular nature of these variants is often inferred based on their phenotypic spectrum relative to individuals with coding LOF variants.

To illustrate the functional impact of mLOE variants, it is helpful to compare the full phenotype associated with coding LOF variants to the modular phenotype associated with mLOE variants in a gene regulatory element for the same gene. For example, coding LOF variants in *GATA1* result in both severe platelet and red blood cell abnormalities, because *GATA1* expression is critical for both of these cell types (Gutiérrez et al. 2020). In contrast, a 4 kb deletion of a megakaryocyte-specific enhancer element for *GATA1* is associated with platelet abnormalities, but normal red blood cell parameters (Turro et al. 2020), as this enhancer is necessary for *GATA1* expression within megakaryocytes but not within red blood cells.

Similarly, whereas coding LOF variants in *PTF1A* cause both pancreatic and cerebellar agenesis (Sellick et al. 2004), deletions or single-nucleotide variants within a pancreas-specific enhancer located 25 kb downstream of *PTF1A* cause only isolated pancreatic agenesis, likely because *PTF1A* expression during cerebellar neurogenesis is maintained (Weedon et al. 2014).

mLOE variants can also be located in promoters. For example, LOF variants in *APC* cause familial adenomatous polyposis, a condition associated with adenocarcinoma and numerous polyps in the stomach and colon. However, APC has two distinct promoters termed 1A and 1B, and *APC* transcription within the stomach mucosa is selectively initiated via promoter 1B. Consequently, individuals with variants in *APC* promoter 1B are at risk for developing gastric adenocarcinoma and proximal polyposis isolated to the stomach (GAPPS) without colon polyposis as a comorbidity (Li et al. 2016).

By selectively disrupting the expression of a gene in only a particular cell type, mLOE variants have the potential to produce a disease phenotype mediated by genes associated with embryonic lethality in the context of coding LOF variants. For example, biallelic LOF variants in *PIGM* are embryonic lethal in mice. In contrast, biallelic variants within the *PIGM* promoter that disrupt an SP1-binding element cause an inherited glycosylphosphatidylinositol deficiency characterized by a propensity for venous thrombosis and seizures (Almeida et al. 2006). The modular phenotype associated with this promoter variant results from the differential importance of this SP1 element in *PIGM* expression across cell types (Costa et al. 2014).

In addition to cell type selectivity, mLOE variants can also cause loss of expression at particular developmental stages. For example, variants that disrupt a C/EBP or HNF4-binding element within the *F9* gene promoter cause Hemophilia B Leyden, which is characterized by severe factor IX deficiency at birth that ameliorates after puberty (Veltkamp et al. 1970). The affected C/EBP- and HNF4-binding elements are essential for *F9* transcription in early childhood. However, after puberty, androgen-responsive TFs bind to an androgen response element within the *F9* promoter, dramatically increasing *F9* transcription to levels that largely resolve the disease phenotype (Crossley et al. 1992). Consequently, Hemophilia B Leyden is caused by a modular loss of *F9* expression only within the prepubescent developmental stage.

In summary, mLOE variants are located within promoter or distal gene regulatory elements, can restrict the disease phenotype associated with coding LOF variants to only a specific tissue or developmental stage, and can result in a disease phenotype for genes wherein coding LOF variants would be embryonic lethal.

## Gain-of-ectopic-expression (GOE) variants

GOE variants cause ectopic spatial and/or temporal expression patterns and represent a disease mechanism that is largely unique to regulatory variants (Table 4). Notably, some GOE variants can mimic Mendelian conditions caused by duplications of the target gene. For example, autosomal-dominant adult-onset demyelinating leukodystrophy (ADLD) is caused by overexpression of LMNB1 protein usually attributed to duplication of the *LMNB1* gene. However, an ADLD family was discovered to have a deletion that begins 66 kb upstream of the *LMNB1* promoter. This deletion encompasses a TAD boundary and results in overexpression of *LMNB1* protein via a mechanism termed ‘enhancer adoption’. Specifically, a strong enhancer that typically does not regulate *LMNB1* is now brought into the same TAD as the *LMNB1* promoter, resulting in *LMNB1* overexpression analogous to that seen with *LMNB1* duplication (Giorgio et al. 2014).

Enhancer adoption is a common mechanism through which structural variants can cause regulatory element GOE (Fig. 1D). For example, structural variants within the *WNT6/IHH/EPHA4/PAX3* locus can cause distinct phenotypes depending on where a strong cluster of limb enhancers for *EPHA4* is situated relative to the *WNT6*, *IHH*, or *PAX3* genes. Specifically, deletion of a TAD boundary between *EPHA4* and *PAX3* results in *PAX3* adopting this cluster of limb enhancers, resulting in ectopic *PAX3* expression and brachydactyly. In contrast, inversions or duplications involving *IHH* and the TAD boundary between *IHH* and *EPHA4* result in *WNT6* adopting this cluster of limb enhancers, resulting in ectopic *WNT6* expression and F-syndrome (Lupiáñez et al. 2015).

SNVs within distal regulatory elements can also cause GOE. For example, the zone of polarizing activity regulatory sequence (ZRS), located in intron 5 of the *LMBR1* gene, regulates *SHH*. SNVs within the ZRS located ~ 1 Mb upstream of *SHH* cause preaxial polydactyly (Lettice et al. 2002; Gurnett et al. 2007; Furniss et al. 2008) via the introduction of novel ETV2-binding sites in the ZRS, resulting in ectopic SHH expression within the developing limb bud (Koyano-Nakagawa et al. 2022). However, it is important to recognize that for a given gene, not all SNVs within distal regulatory elements result in the same phenotype, as non-coding SNVs within the *SHH* brain enhancer-2 (SBE2) located 460 kb upstream of *SHH* cause holoprosencephaly via an LOE mechanism (Jeong et al. 2008).

In addition to distal regulatory elements, GOE variants can also affect promoters. For example, glucocorticoid-remediable aldosteronism (GRA) is caused by ‘promoter switching’ between the genes *CYP11B1* and *CYP11B2*, resulting in a chimeric gene wherein the adrenocorticotropic hormone (ACTH)-responsive promoter of the 11-beta-hydroxylase gene (*CYP11B1*) is fused with the coding region of the aldosterone synthase gene (*CYP11B2*) (Lifton et al. 1992). This results in ectopic expression of aldosterone synthase in zona fasciculata cells of the adrenal cortex, causing aldosterone synthase to be overexpressed and inducible by ACTH, hence a hyperaldosteronism state that normalizes upon treatment with glucocorticoids.

GOE variants can also cause Mendelian conditions for which the target gene does not have a known human phenotype associated with coding LOF variants, such as when coding LOF variants would result in embryonic lethality. This is notable, because the clinical identification of non-coding variants that cause Mendelian conditions is often informed by comparison to known LOF phenotypes. For example, complete loss of *OVOL2* expression has been associated with embryonic lethality in mice, likely because OVOL2 is a transcription factor critical for epithelial cell lineage determination and differentiation (Mackay et al. 2006). Meanwhile, in humans, *OVOL2* promoter variants that result in GOE can cause autosomal-dominant corneal endothelial dystrophies. These promoter variants result in the creation of binding elements for several activating TFs within the *OVOL2* promoter, resulting in the inappropriate ectopic expression of *OVOL2* in the developing or adult corneal endothelium (Davidson et al. 2016).

Promoter GOE variants can also disrupt the ability of transcriptional repressors to appropriately silence a gene at a particular developmental stage. For example, the gamma-globin genes *HBG1* and *HBG2* encode a component of fetal hemoglobin (HbF) and are normally expressed only during fetal erythropoiesis, as their promoters are silenced during adult erythropoiesis by the transcriptional repressors BCL11A and ZBTB7A. However, regulatory variants within the *HBG1* and *HBG2* promoters that disrupt BCL11A- and ZBTB7A-binding elements result in the hereditary persistence of fetal hemoglobin (HPFH) into adulthood (Martyn et al. 2018). As HbF is capable of preventing red blood cell sickling from sickle hemoglobin (HbS) and can compensate for deficient HbA as seen in beta-thalassemia, these HPFH variants can attenuate the phenotype of sickle cell disease and beta-thalassemia (Jackson et al. 1961; Cappellini et al. 1981; Labie et al. 1985; Weatherall 2001; Thein 2008; Thein et al. 2009). The discovery of HPFH variants has fortuitously enabled the development of gene editing therapies, which introduce these variants into adult erythroid progenitor cells to reactivate HbF as treatment for sickle cell disease and beta-thalassemia (Traxler et al. 2016; Li et al. 2021).

GOE variants and LOE variants impacting the same gene can lead to similar phenotypes. For example, a “Goldilocks” level of *FOXG1* expression is likely required for normal brain development, because both *FOXG1* duplications and deletions are associated with Rett-like phenotypes (Florian et al. 2012). Thus, it is unsurprising that GOE variants that remove a silencer and LOE variants that remove an enhancer have both been reported to cause Rett-like phenotypes via increasing and decreasing *FOXG1* expression, respectively (Kortüm et al. 2011; Allou et al. 2012).

Finally, variants within gene regulatory elements can cause mixed effects. For example, the *POMP* gene typically has a short 5’UTR that originates from a TSS located at position c.-81. A single-nucleotide deletion in the *POMP* promoter at position c.-95 does not change the overall transcript levels of *POMP*, but results in decreased utilization of the canonical TSS and increased utilization of an upstream TSS located at position c.-181. This results in *POMP* transcripts that preferentially contain a long 5’UTR with reduced translational efficiency. Consequently, *POMP* expression within the granular layer of the epidermis is reduced, causing keratosis linearis with ichthyosis congenita and sclerosing keratoderma (KLICK) syndrome (Dahlqvist et al. 2010). This example illustrates how non-coding variants can have mixed effects, wherein they result in GOE of one transcript, LOE of a different transcript, and LOF at the protein level. In contrast, coding GOF variants in *POMP* result in proteasome-associated autoinflammatory syndrome 2 (PRAAS2) which has a quite distinct clinical presentation (Poli et al. 2018), demonstrating that gene regulatory GOE and coding GOF variants involving the same gene can cause completely different clinical phenotypes.

In summary, GOE can result from structural variants and SNVs located within promoters and distal gene regulatory elements. GOE variants often arise from the ectopic activity of enhancers (e.g., enhancer adoption) or promoters, or the disruption of normal repressive gene regulatory machinery. Furthermore, variants can cause complex gene regulatory outcomes wherein they cause GOE for one transcript, but LOE for a different one. Importantly, GOE variants often result in clinical phenotypes that markedly diverge from that of coding variants, complicating efforts to systematically identify this class of genetic variation using our current catalog of phenotypes associated with coding variants.

## Concluding thoughts

In this review, we summarize the literature on gene regulatory variants that are known to cause Mendelian conditions and present a framework for categorizing these variants based on their proximate impact on gene expression patterns. We highlight that certain classes of gene regulatory variants can mimic coding LOF variants and gene duplication variants. However, gene regulatory variants can also create novel phenotypes. Specifically, the phenotypes associated with GOE and mLOE variants may markedly differ from those associated with LOE or LOF variants impacting the same gene. Consequently, extrapolating our knowledge of coding variants to the other 99% of the genome is insufficient for resolving how variants within gene regulatory elements cause Mendelian conditions. The current practice for identifying non-coding variants that cause Mendelian conditions often relies upon the phenotypic similarity to known coding LOF phenotypes, delaying or missing the identification of non-coding genetic variants when the resulting phenotype differs substantially from coding LOF of the same target gene. A functional classification system tailored to the impact of non-coding variants can facilitate the organization of knowledge, so that novel non-coding variants are more readily identified. Additionally, this functional classification system has the potential to improve how we integrate results from regulatory element mutational scanning experiments with observed genetic variants in databases like ClinVar. Specifically, this functional framework can serve as a standardized framework to articulate the functional impact of non-coding variants in relation to different disease phenotypes.

**Fig. 2:**
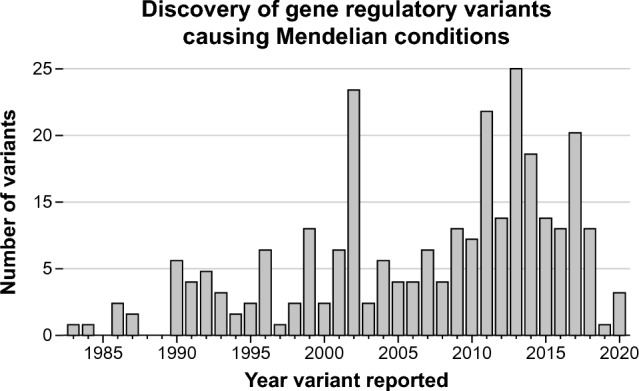
Pace of discovery of gene regulatory variants causing Mendelian conditions. Histogram of the year of publication for all variants cited in this manuscript

Although it has been well established for several decades that gene regulatory variants cause numerous Mendelian conditions in a dominant, recessive, or X-linked inheritance pattern, our current catalog of disease-causing variants is overwhelmingly populated with coding variants. Specifically, whereas ClinVar contains over 150,000 pathogenic or likely pathogenic coding variants (Landrum et al. 2018), our non-systematic review of the literature identified only several hundred genetic variants known to disrupt gene regulatory elements (Fig. 2). It is possible that this imbalance accurately reflects the relative contributions of coding and gene regulatory variants to Mendelian conditions. However, it is notable that the rate of discovery of non-coding regulatory variants has only modestly increased since the transition in 2010 from family-based linkage analysis to exome sequencing as the predominant mode for gene discovery and clinical testing (Fig. 2). In contrast, the rate of discovery of pathogenic coding variants has substantially increased over the past 10 years (Landrum et al. 2018; Bamshad et al. 2019). Consequently, we hypothesize the current imbalance in the identification of pathogenic coding variants over gene regulatory variants more likely reflects the inadequacy of exome sequencing and current tools for analysis and interpretation to implicate this class of variation in disease. As the use of genome sequencing and epigenetic profiling becomes more common within clinical genomics, we anticipate that more examples of gene regulatory variation causing Mendelian conditions will emerge.

## Data Availability

No new data were created or analyzed in this study.
